# Green synthesis of purple *sweet potato*-derived selenium nanoparticles accelerates wound healing through pyroptosis regulation

**DOI:** 10.1016/j.mtbio.2025.102269

**Published:** 2025-09-02

**Authors:** Chen Chen, Fructueux Modeste Amona, Ziqi Sha, Jiamin Li, Yongding Ke, Yuxin You, Luyuan Yang, Guangfu Liao, Xi Chen, Yipeng Pang, Yi Liu

**Affiliations:** a College of Hydraulic Engineering, Jiangsu Vocational Institute of Architectural Technology, Xuzhou, 221000, Jiangsu, China; b Institute of Cellular and Molecular Biology, School of Life Science, Jiangsu Normal University, Xuzhou, 221116, Jiangsu, China; c Department of Biophysics, School of Life Sciences, Xuzhou Medical University, Xuzhou, 221004, Jiangsu, China; d College of Material Engineering, Fujian Agriculture and Forestry University, Fuzhou, 350002, China

**Keywords:** Selenium nanoparticles, Purple *sweet potato*, Green synthesis, Antimicrobial, Pyroptosis, Wound healing

## Abstract

Although advances in nanomedicine using nanoparticles (NPs) derived from natural compounds have provided us with much insight into how to recover from wound infections, exploring the wound healing pathway remains a new perspective that has attracted significant interest in addressing wound complications challenges. Here, we harnessed the therapeutic potential of novel selenium nanoparticles (SeNPs) derived from Purple *sweet potato* (PSp) extracts to promote wound healing through the regulation of pyroptosis-related pathways. PSp-SeNPs, with an average particle size of 80–100 nm, demonstrated significant antibacterial activity against *S. aureus* and MRSA clinical pathogens. The mechanism involves impairment of bacterial growth, biofilm formation, and metabolic processes through ATP depletion. Moreover, PSp-SeNPs impairs NLRP3-mediated pyroptosis including p-IκBα, p-NF-κB, IL-18, and IL-1β. This regulatory effect decreases inflammatory cytokines IL-6 and TNF-α while promoting angiogenesis and collagen formation through increased expression levels of TGF-β, VEGFA, and CD31, thus accelerating wound healing. *In vivo* assessments confirmed that PSp-SeNPs significantly enhanced wound healing without adverse effects, indicating their high biocompatibility and bioavailability. This groundbreaking study elucidates the therapeutic potential of PSp-based selenium nanoparticles, facilitating the development of precise and efficient treatment strategies for wound healing and diverse medical applications.

## Highlights

• PSp-SeNPs as a targeted therapeutic approach for boosting wound healing.
• PSp-SeNPs show excellent antibacterial activity against *S. aureus* and MRSA.
• PSp-SeNPs inhibit NF-κB, which affects the NLRP3-mediated pyroptosis and decreases IL-18 and IL-1β.
• PSp-SeNPs enhance angiogenesis and collagen production.
• PSp-SeNPs significantly improve wound healing while showing no adverse effects.

## Graphical abstract

**Figure:**
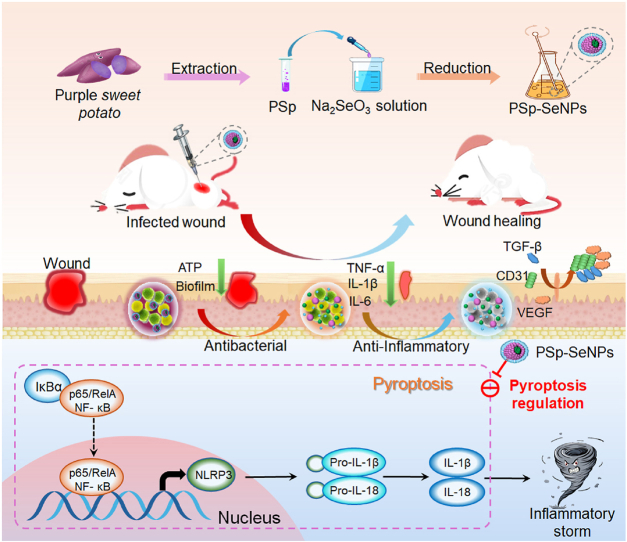

## 1 Introduction

Wound healing constitutes a dynamic cascade of coordinated biological events, progressing through four interdependent stages: hemostatic response, inflammatory regulation, tissue regeneration, and matrix maturation [1,2]. Inadequate or impaired wound healing is commonly induced by gram-positive bacteria, such as *Staphylococcus aureus* (*S. aureus*), especially its β-lactam-derived antibiotic-resistant strains, methicillin-resistant *S. aureus* (MRSA), leading to severe or chronic wound infections [[3], [4], [5]]. These complications pose substantial healthcare issues and weigh on economies globally [[6], [7], [8]]. Pyroptosis is a programmed cell death pathway stimulated by microbial infections, releasing pro-inflammatory cytokines and various molecular patterns that aid the immune response [[9], [10], [11]]. In wound infections, this process is activated through the nucleotide-binding oligomerization domain-like receptor (NLR) family pyrin domain-containing 3 (NLRP3)/caspase-1 route, which contributes to prolonged inflammation [[12], [13], [14]]. Recent research suggests that increased NLRP3 levels in diabetic individuals might contribute to chronic inflammation and oxidative stress, thereby impairing wound healing [15,16]. Inhibiting NLRP3 inflammasome activation emerges as a novel therapeutic paradigm for dual modulation of pyroptotic pathways and accelerated tissue regeneration in wound repair.

Recent advances in biobased nanotechnology have introduced nanoparticles (NPs) derived from natural compounds that enhance wound healing through antibacterial, anti-inflammatory, and angiogenic properties [[17], [18], [19], [20]]. Selenium nanoparticles (SeNPs) show particular promise due to their multifunctional benefits, including antioxidant and antimicrobial properties [19,21], higher bioavailability, and lower toxicity compared to ionic selenium (Se) [[22], [23], [24]]. However, current SeNP-based therapies are limited by the cytotoxicity and poor biocompatibility of chemically synthesized SeNPs [23,25], the lack of targeted pyroptosis regulation in plant-derived SeNPs that affects their efficacy in addressing chronic inflammation [26,27], and the challenges in scaling and reproducing green synthesis methods due to instability in plant reductants [28,29].

To address these significant gaps, SeNPs derived from Purple *sweet potato* extracts (PSp-SeNPs) were engineered for accelerated wound healing. Purple *sweet potato* **(**Psp), known as *Ipomoea batatas*, is a perennial herb from the *Convolvulaceae* family and a promising natural source for extracting SeNPs owing to its high nutritional value and health benefits [30,31]. It is rich in bioactive compounds, including polysaccharides, proteins, high levels of anthocyanins and phenolic compounds, demonstrating significant antioxidant and anti-inflammatory properties [32,33]. PSp-SeNPs uniquely provide three advantages over previous SeNP-based natural compounds [28,29,34]. The polyphenolic compositions of PSp ensure high reducing power and colloidal stability, enabling NPs optimized for bacterial membrane penetration. PSp bioactive synergizes with Se to concurrently target NLRP3-mediated pyroptosis and bacterial virulence factors. Finally, their green synthesis is scalable and prevents toxic residues.

**Scheme 1:**
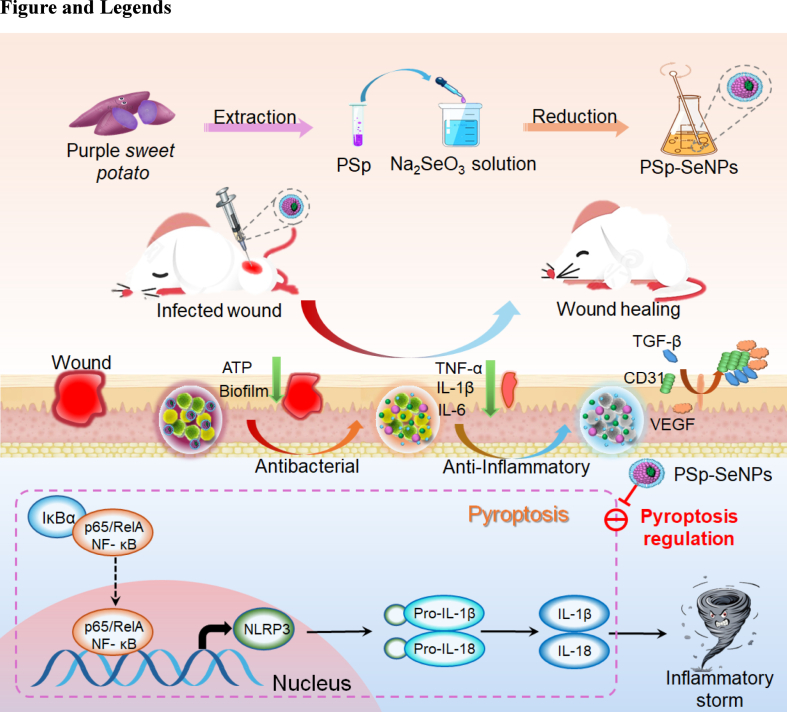
Schematic diagram illustrating the mechanism by which SeNPs synthesized from PSp extracts enhance wound healing through antimicrobial properties and pyroptosis regulation.

However, to our knowledge, no research has comprehensively explored the function of PSp-SeNPs as pyroptosis regulators in infected wound models. Thus, we investigated the therapeutic potential of PSp-SeNPs to enhance wound healing by regulating pyroptosis (Scheme 1). PSp-SeNPs were optimized and characterized via ultraviolet–visible (UV–Vis) spectroscopy, X-ray photoelectron spectroscopy (XPS), transmission electron microscopy (TEM), scanning electron microscopy (SEM), and X-ray diffraction (XRD). Interestingly, PSp-SeNPs significantly inhibited clinical pathogens *S. aureus* and MRSA. Furthermore, they promoted wound healing by regulating pyroptosis. Additionally, the *in vivo* wound-healing activity of the PSp-SeNPs significantly accelerated wound healing. This work establishes PSp-SeNPs as a biocompatible, multi-mechanistic nanotherapy that overcomes key limitations of existing SeNP platforms.

## 2 Materials and methods

### 2.1 Preparation of purple *sweet potato* Extract

As previously described [30,31], solvent extraction was used as the primary method for obtaining the PSp extracts. Briefly, to prepare the purple *sweet potato* extract, 5 g of purple *sweet potato* extract powder was dissolved in 100 mL of H_2_O. The mixture is stirred constantly for 30 min to release bioactive compounds from the powder into the solution. Afterward, the solution was filtered through Whatman No. 1 filter paper (pore size 11 μm) to remove residual solid particles, yielding a clear purple extract. No centrifugation was performed before filtration. This freshly prepared extract is then used immediately to synthesize selenium nanoparticles (PSp-SeNPs), ensuring the retention of the bioactive compound.

### 2.2 Preparation and synthesis of PSp-SeNPs

PSp-SeNPs were synthesized as previously described [23,28,35] with some modifications as follows. PSp-SeNPs were synthesized by mixing 50 mL of the purple sweet potato extract solution with 100 mL of a 5 mM Na_2_SeO_3_ solution. The mixture was stirred for 2 h, resulting in a color change from colorless to reddish-brown, suggesting the formation of PSp-SeNPs [36]. The optimal synthesis conditions were identified as a pH of 8, with adjustments made via 0.1 M NaOH or HCl and a reaction time of 2 h to produce stable and uniform nanoparticles. After synthesis, the PSp-SeNPs were purified by centrifugation at 10,000 rpm for 15 min and then washed three times with H_2_O to thoroughly eliminate unnecessary components. The purified PSp-SeNPs were then freeze-dried to acquire a dry powder.

### 2.3 Antimicrobial susceptibility testing *in vitro*

The minimum inhibitory concentration **(**MIC) of PSp-SeNPs was tested against *S. aureus* and MRSA via a broth microdilution assay as previously described [37,38]. Serial twofold dilutions of PSp-SeNPs (starting at 2048 μg/mL) were prepared in test tubes containing nutrient broth. The inoculum (1 × 10^8^ CFU/mL) was grown overnight in the nutrient broth at 37 °C for 18–24 h. The lowest PSp-SeNP concentration at which no discernible bacterial growth was seen was known as the minimum inhibitory concentration, or MIC.

### 2.4 Bacteria count assay

A bacterial count assay was performed to determine the influence of PSp-SeNPs on bacterial viability. After PSp-SeNPs treatment, representative aliquots (1 × 10^8^ CFU/mL) from experimental cohorts were aseptically inoculated onto nutrient agar plates, followed by controlled incubation at 37 °C for an 18–24 h duration. Colony growth was monitored through photographs, and to evaluate bacterial survival, the quantity of colony-forming units (CFUs) was measured.

### 2.5 Resazurin staining test

Resazurin staining was used to evaluate bacterial viability after PSp-SeNPs treatment. Resazurin solution was introduced to each 96-well plate, including the treated bacterial cultures, and incubated at 37 °C for 4 h. A visual inspection revealed a color change from blue (oxidized) to pink (reduced), reflecting the metabolic activity of the bacteria. A greater color indicates greater bacterial viability, whereas a minimal color change suggests reduced viability owing to PSp-SeNP treatment.

### 2.6 Bacterial biofilm clearance test

Crystal violet staining was utilized to evaluate bacterial biofilm formation following PSp-SeNPs treatment. The bacterial cultures were transferred to 96-well plates and incubated at 37 °C for 24 h to promote biofilm formation. The wells were then gently washed with PBS to eliminate nonadherent bacteria before staining the biofilms with a 0.1 % crystal violet solution for 15 min. Excess dye was washed through PBS, and the biofilm-bound crystal violet was solubilized with 95 % C_3_H_5_OH. The absorbance was measured at 600 nm via a microplate reader as previously described [39]. The optical density (OD) was used to quantify the biofilm biomass and assess the effect of PSp-SeNPs on biofilm formation.

### 2.7 ATP level detection in bacteria

An ATP assay kit (HY-K0314, MCE, China) was used to measure adenosine triphosphate (ATP) levels in bacterial cells after treatment with PSp-SeNPs. Bacterial cultures were collected, washed with PBS, and then resuspended in lysis buffer at room temperature for 5 min to release intracellular ATP. The bacterial lysate (50 μL) was mixed with ATP detection reagent in a 96-well plate, and luminescence was determined via a microplate reader as previously described [40]. The light intensity correlated with the ATP concentration, indicating the metabolic activity and viability of PSp-SeNPs after treatment.

### 2.8 Monitoring the growth curve of bacteria

A 96-well plate was prepared with 200 μL of Mueller‒Hinton broth (MHB) and 100 μL of bacterial inoculum (1 × 10^8^ CFU/mL). PSp-SeNPs were added to the wells, with PBS serving as the control. Following incubation at 37 °C under controlled conditions, bacterial proliferation was dynamically quantified through spectrophotometric analysis at 600 nm using a microplate reader.

### 2.9 Wound healing assessment *in vivo*

All animal protocols followed the National Institutes of Health's instructions for the Care and Use of Laboratory Animals, which were approved by the Animal Ethics Committee of Jiangsu Normal University and assigned the study ethics number [JSNU-IACUC 2024073].

Twenty-four male Kunming mice (Mus musculus; 6–8 postnatal weeks, 25–30 g body weight) underwent standardized acclimatization in polypropylene enclosures with ad libitum feeding protocols under controlled vivarium conditions: 25 °C ambient temperature, 55 ± 5 % relative humidity, and 12 h/12 h photoperiodic regulation. The mice were randomly divided into four groups (n = 6 per group): group 1 (control, PBS), group 2 (PSp extract), group 3 (Na_2_SeO_3_), and group 4 (PSp-SeNPs). The skin was excised utilizing an 8 mm diameter punch biopsy. To minimize skin retraction and ensure reproducible imaging, the mice were gently positioned, and the wound area was exposed with minimal tension before photographing. A fixed distance and angle were maintained, and a scale bar was included for calibration. The wound was then inoculated with S. aureus for three days to establish a model of wound infection. After inoculation, the presence of bacteria was confirmed through the macroscopic detection of infectious secretions (photodocumented) and cultivation [41,42]. PSp extract, Na_2_SeO_3_, and PSp-SeNPs were applied topically at a concentration of 256 μg/mL, expressed as the total mass of the complex for PSp-SeNPs, which contains approximately 40–50 % selenium and 50–60 % PSp-derived bioactives by weight. These treatments were applied once daily to the wounds for 7 days, while the control group received PBS. Weights and infected wound sizes were measured to assess healing effectiveness. Post-interventional euthanasia via cervical dislocation (AVMA compliant) was performed, with subsequent aseptic necropsy to harvest lesion specimens and core organs (cardiac, hepatic, splenic, pulmonary, renal) for ultra-low temperature cryopreservation (−80 °C). Wound tissues from three randomly selected mice per group were subjected to Western blotting, ELISA, and RT‒qPCR, and the remaining tissues were harvested for histopathological analysis.

### 2.10 Survival determination of bacteria in wounds

Post-PSp-SeNPs administration, dermal lesion specimens were aseptically excised with sterile surgical blades in ice-cold phosphate-buffered saline (pH 7.4), followed by 5 min low-speed vortex agitation under laminar airflow conditions. These samples were diluted and cultured on Muller-Hinton agar (MHA) plates for 24 h at 37 °C. The impact of PSp-SeNPs was assessed by quantifying colonies to determine bacterial load in the wounds, as previously described [43,44].

### 2.11 Evaluation of hemolytic activity

The potential of PSp-SeNPs to lyse red blood cells was assessed using a standard hemolysis assay. Briefly, 0.5 mL of murine whole blood was centrifuged (3500 rpm, 5 min) to isolate erythrocytes. The erythrocyte pellet underwent three washes with sodium thiobarbital solution to prepare a 5 % (v/v) suspension. Aliquots of this suspension were mixed with PSp-SeNPs at concentrations of 50, 100, and 200 μg/mL and incubated with gentle agitation (100 rpm) for 1 h at 37 °C. Following incubation, the mixtures were centrifuged (3500 rpm, 5 min), and hemoglobin release into the supernatant was quantified by measuring absorbance at 545 nm. PBS and deionized water served as negative (0 % hemolysis) and positive (100 % hemolysis) controls, respectively. The percentage hemolysis was calculated as follows:

$$\text{Hemolysis}\;\text{ratio}\;\left(\%\right)=\frac{{\text{OD}}_{\text{sample}}-{\text{OD}}_{\text{PBS}}}{{\text{OD}}_{\text{water}}-{\text{OD}}_{\text{PBS}}}\times 100\%\text{.}$$

OD_sample_ represents the value for erythrocytes exposed to different concentrations of PSp-SeNPs, respectively.

### 2.12 Blood biochemical evaluation

Systemic toxicity of PSp-SeNPs was further assessed by blood biochemistry, emphasizing liver and kidney injury markers. Blood was drawn from mice treated with PSp-SeNPs (4 mg/kg). Serum was separated by centrifugation (3000 rpm, 10 min, 4 °C) and analyzed for concentrations of alanine aminotransferase (ALT), aspartate aminotransferase (AST), blood urea nitrogen (BUN), and creatinine (CRE). These analyses were conducted using validated commercial kits (Nanjing Jiancheng, China) following the supplier's instructions.

### 2.13 Cellular viability assay

To assess the cytotoxic effects of PSp-SeNPs, human umbilical vein endothelial cells (HUVECs) were cultured in 96-well plates at a density of 1 × 10^4^ cells/well in 100 μL DMEM supplemented with fetal bovine serum (FBS) and antibiotics (penicillin and streptomycin). Cells were exposed to PSp-SeNPs and maintained under standard culture conditions (37 °C, 5 % CO_2_) for 24 h. Post-treatment, cellular viability was quantified using a Cell Counting Kit-8 (CCK-8) colorimetric assay.

## 3 Results and discussion

### 3.1 Synthesis and characterization of PSp-SeNPs

Recent advances in processing technologies have streamlined the extraction of active plant components, decreasing extraction times and enhancing product quality. While solvent extraction remains the common method for extracting PSp [30,31], selecting the optimal technique is crucial. This choice ensures that functional activity is maximized, production is feasible, and undesirable property variations are minimized. SeNPs effectively kill pathogenic bacteria, particularly in chronic infections. For the environmentally eco-friendly synthesis of SeNPs, widely accessible medicinal plants such as PSp are used [29]. Chosen for their pharmacological activity and reducing/stabilizing properties [30,45,46], PSp's polyphenols also prevent aggregation and yield smaller particles during green synthesis [29].

**Fig. 1:**
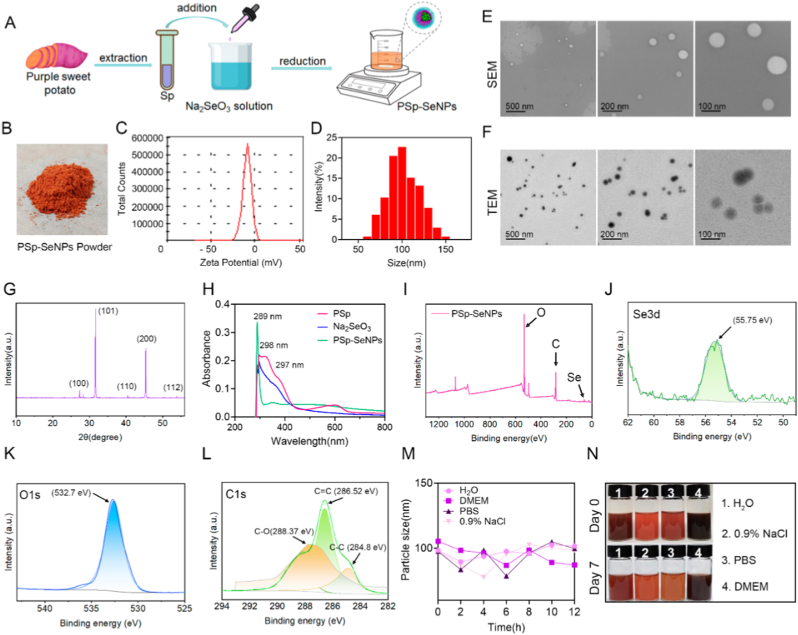
Preparation and characterization of PSp-SeNPs. (A) Schematic diagram illustrates the green synthesis of PSp-SeNPs. (B) Visual representation of the PSp-SeNPs powder. (C) Zeta potential analysis of the PSp-SeNPs. (D) Particle size of PSp-SeNPs. (E) SEM images depict the morphology of PSp-SeNPs. Scale bar: 500 nm, 200 nm, and 100 nm. (F) TEM images of PSp-SeNPs. Scale bar: 500 nm, 200 nm, and 100 nm. (G) XRD pattern of the PSp-SeNPs. (H) UV–Vis absorbance spectrum of PSp, Na_2_SeO_3_, and PSp-SeNPs. (I) XPS analysis of PSp-SeNPs with peaks corresponding to oxygen (O), carbon (C), and selenium (Se). (J–L) High-resolution XPS spectrum of PSp-SeNPs with chemical states corresponding to Se3d (J), O1s (K), and C1s (L). (M) Time-dependent analysis of PSp-SeNPs in H_2_O, DMEM, PBS, and 0.9 % NaCl over 12 h. (N) Visual images of PSp-SeNPs on Day 0 and Day 7 in H_2_O, 0.9 % NaCl, PBS, and DMEM. (For interpretation of the references to color in this figure legend, the reader is referred to the Web version of this article.)

In this study, we used solvent extraction methods to obtain PSp extracts and green-synthesized PSp-SeNPs nanomedicine agents (Fig. 1A). The preparation of SeNPs was observed by the brick-red color following extraction with a Na_2_SeO_3_ reaction (Fig. 1B). DLS analysis showed that the PSp-SeNPs had a predicted zeta potential of −13.5 mV (Fig. 1C) and an average particle size of 90.1 nm (Fig. 1D), suggesting good stability without aggregation. SEM and TEM indicated that the nanoparticles were shaped as spherical and monodispersed with approximately 80–100 nm in size (Fig. 1E and F). The XRD pattern showed the formation of nanocrystalline PSp-SeNPs (Fig. 1G), which matched the standard selenium powder. Characteristic diffraction peaks were noted at 23.11°, 31.66°, 40.46°, 45.39°, and 53.82°, corresponding to the (100), (101), (110), (200), and (112) lattice planes of hexagonal selenium, respectively, aligning well with the standard reference (PDF65-1876) [35]. Research on plant extract-derived SeNPs has reported similar results [25,34].

The UV–Visible absorption spectrum of the PSp-SeNPs dispersion displayed a maximum absorbance peak at 289 nm, whereas the wavelengths of the PSp and Na_2_SeO_3_ dispersions shifted to 298 and 297 nm, respectively (Fig. 1H). This result is consistent with earlier research on UV–Visible spectroscopy of SeNPs [25,36,42]. X-ray photoelectron spectroscopy (XPS) analysis revealed the presence of selenium (Se), oxygen (O), and carbon (C) (Fig. 1I), corresponding to the binding energies of Se 3d (Fig. 1J), O1s (Fig. 1K), and C1s (Fig. 1L), respectively. The strong peaks observed at 55.75, 532.7, and 286.52 eV in the XPS spectra provided typical evidence that the PSp-SeNPs consisted of Se, O, and C organic components, respectively. The spatial distribution of Se in the PSp-SeNPs was confirmed by the Se 3d peak at 55.75 eV, demonstrating its incorporation through green nanobiosynthesis. High-resolution C1s spectra revealed peaks at 284.80 (C=C), 286.52 (C–C), and 288.37 eV (C–O), consistent with previously reported values (Fig. 1L) [47]. Finally, investigations into the particle size stability of PSp-SeNPs across various solutions showed that H_2_O and 0.9 % NaCl provide stable conditions with no significant size changes (Fig. 1M and N).

Collectively, this study used PSp extract as a renewable reducing and stabilizing agent in a green synthesis approach, reducing the need for harsh chemicals and aligning with eco-friendly principles. However, large-scale cultivation must prioritize sustainable agricultural practices to avoid land-use conflicts and excessive water consumption. Energy-efficient optimization of extraction and synthesis steps, along with responsible management of organic by-products, is essential to minimize ecological impact. Future work should include life cycle assessments to quantify resource use, emissions, and waste generation.

### 3.2 Antimicrobial activity of PSp-SeNPs

**Fig. 2:**
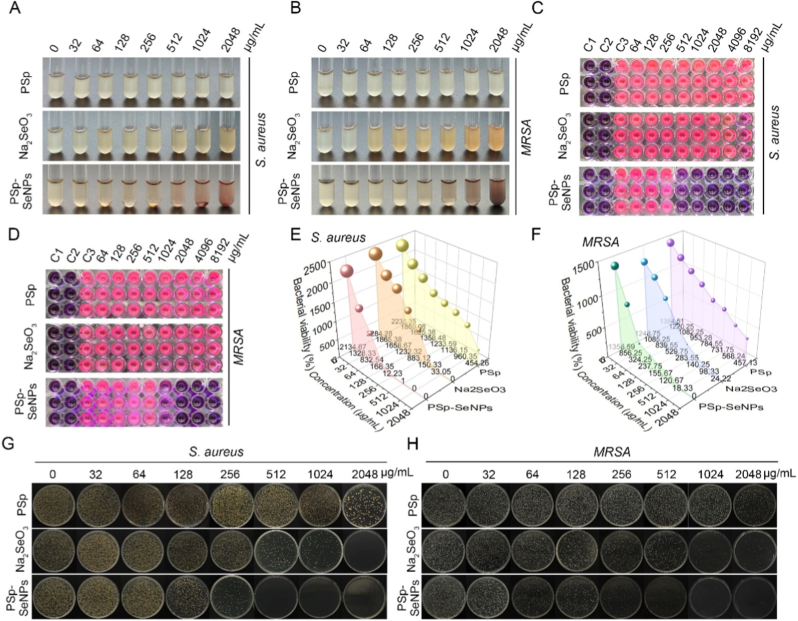
Antimicrobial activity of PSp-SeNPs. (A–B) Culture tubes showing the effects of PSp, Na_2_SeO_3_, and PSp-SeNPs treatments on *S. aureus* (A) and MRSA (B). (C–D) Resazurin staining of *S. aureus* (C) and MRSA (D) growth after PSp, Na_2_SeO_3_, and PSp-SeNPs treatments. (E–F) Quantification of *S. aureus* (E) and MRSA (F) growth after treatments with PSp, Na_2_SeO_3_, and PSp-SeNPs. (G–H) Growth plating assays of *S. aureus* (G) and MRSA (H) following treatment with PSp, Na_2_SeO_3_, and PSp-SeNPs.

Plant extract-derived SeNPs are potential antimicrobial agents used in medical settings to treat microbial infections [19,48]. To investigate this, we also analyzed the antibacterial activity of PSp-SeNPs against significant clinical bacterial pathogens *S. aureus* and MRSA using the micro-dilution assay. The results showed increasing MICs for PSp-SeNPs, with significant inhibition of *S. aureus* (Fig. 2A) at an MIC of 128 μg/mL, and MRSA (Fig. 2B), with an MIC of 512 μg/mL, compared with those of Na_2_SeO_3_ and PSp (Table 1), highlighting their effective antibacterial activity at low MICs. Resazurin staining showed greater inhibition of *S. aureus* (Fig. 2C) and MRSA (Fig. 2D) by PSp-SeNPs than by Na_2_SeO_3_ or PSp, as demonstrated by the color changes (purple or pink hues) in the microplates. Quantitative analyses and colony counts further confirmed the reduced growth of *S. aureus* (Fig. 2E and G) and MRSA (Fig. 2F and H) after treatment with PSp-SeNPs. Consistently, several studies on natural compound extract-derived SeNPs have reported similar results [49]. Huang et al. synthesized selenium-conjugated nanoparticles with quercetin and acetylcholine to evaluate their effectiveness against multidrug-resistant superbugs, including *Escherichia coli* and *S. aureus* [50]. Phytofabrication of SeNPs from *Emblica officinalis* fruit extract (PF-SeNPs) has demonstrated potent antibacterial activity against various pathogens, such as *S. aureus* and MRSA [51]. Recently, it has been shown that SeNPs derived from *Amphipterygium glaucum* extract have antibacterial activity against *S. marcescens, A. faecalis, and E. cloacae* strains [34].

**Table 1:** The minimum inhibitory concentration (MIC) of PSp, Na_2_SeO_3,_ and PSp-SeNPs against *S. aureus* and *MRSA*.

Strains	PSp (μg/mL)	Na_2_SeO_3_ (μg/mL)	PSp-SeNPs (μg/mL)
*S. aureus*	>2048	1024	128
*MRSA*	>2048	2048	512

The antimicrobial efficacy of PSp-SeNPs (MIC: 128 μg/mL for *S. aureus*, 512 μg/mL for MRSA) compares favorably to classical chemically synthesized SeNPs. For instance, SeNPs synthesized using sodium borohydride have been reported to exhibit MICs against *S. aureus* ranging from 200 to 500 μg/mL [52], while other chemical methods often result in even higher MIC values or require surface functionalization to achieve significant activity [23]. The superior performance of PSp-SeNPs is attributed to their natural biofunctionalization with PSp polyphenols, which enhances membrane penetration and synergistic antibacterial action, overcoming a key limitation of bare chemical SeNPs. Collectively, these discoveries indicate the potential of PSp-SeNPs in the development of novel nanomedicines for treating bacterial infections, particularly in cases of antibiotic resistance.

**Fig. 3:**
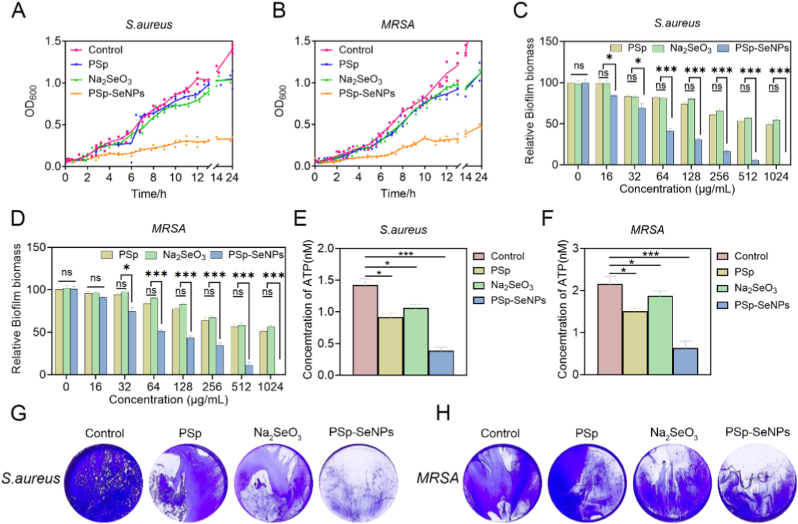
Antimicrobial assessment of PSp-SeNPs treatment. (A–B) Growth curves of PSp, Na_2_SeO_3_, and PSp-SeNPs treatments on *S. aureus* (A) and MRSA (B) growth. (C–D) Biofilm inhibition of *S. aureus* (C) and MRSA (D) following PSp, Na_2_SeO_3_, and PSp-SeNPs treatments. (E–F) Quantification of ATP levels after PSp, Na_2_SeO_3_, and PSp-SeNPs treatment on *S. aureus* (E) and MRSA (F) growth. (G–H) Representative crystal violet staining images showing biofilm formation of *S. aureus* (G) and MRSA (H) after treatment with PSp, Na_2_SeO_3_, and PSp-SeNPs at 256 μg/mL. Data represent mean ± SD (n = 3), with different significance *∗p < 0.05, ∗∗p < 0.01, ∗∗∗p < 0.001.* (For interpretation of the references to color in this figure legend, the reader is referred to the Web version of this article.)

Extracts of natural compounds SeNPs limit the formation of biofilms and depolarize and rupture the bacterial membrane, among other antibacterial processes [53]. Biofilms are a key mechanism in the pathogenicity of bacteria in humans, protecting against antibiotics and host defense mechanisms [54]. Thus, inhibiting the biofilms could be a useful strategy to minimize their pathogenicity. Research has shown that NPs can penetrate and disrupt the biofilm water channels [39]. Consistent with this finding, we first investigated the potential of PSp-SeNPs to disrupt bacterial growth and found that compared with treatment with Na_2_SeO_3_, treatment with various concentrations of PSp-SeNPs restrained the growth of *S. aureus* (Fig. 3A) and MRSA (Fig. 3B), which are similar to previous research [53], where the application biosynthesized of SeNPs from *C. officinalis* extracts against *S. marcescens* inhibited the strain after 2 h of incubation. Similarly, Menon et al. [55] reported that SeNPs (size >100 nm) significantly inhibited bacterial growth after 5 h of incubation. Furthermore, after 24 h of culture incubation to obtain biofilms, the results revealed that compared with Na_2_SeO_3_ and PSp, PSp-SeNPs at a concentration of 256 μg/mL significantly reduced biofilm formation in *S. aureus* (Fig. 3C) and MRSA (Fig. 3D). This result is consistent with previous research, which showed antibiofilm efficacy against pathogens including *Bacillus cereus, Enterococcus faecalis, S. aureus, and E. coli* [29,39,56].

Notably, adenosine triphosphate (ATP) serves as the fundamental energy currency in cellular processes, driving metabolic activities and biosynthesis [57]. Its depletion has been linked to the antibacterial mechanism of natural compound extract-derived SeNPs [51,58]. To further understand this mechanism, we assessed the ATP levels in *S. aureus* and *MRSA* after treatment with PSp, Na_2_SeO_3_, and PSp-SeNPs. We found that, compared with Na_2_SeO_3_ and PSp, PSp-SeNPs significantly decreased the ATP concentration in both *S. aureus* (Fig. 3E) and MRSA (Fig. 3F). These results suggest that PSp-SeNPs impaired bacterial metabolic processes, leading to energy depletion and inhibition of bacterial growth. Nanoparticle size significantly influences antibacterial activity, with optimal effects observed for particles smaller than 100 nm [59]. In this study, PSp-SeNPs-sized 80–100 nm offered a larger contact surface area, facilitating easier penetration through bacterial cell walls and membranes, which cause cell lysis, disrupt ATP synthesis, and impair cell division, ultimately leading to bacterial death [51]. According to Serov et al. [60], the antibacterial mechanisms of SeNPs involve protein degradation caused by the slow release of selenium ions, which interact with the -SH, -NH, or -COOH of proteins and enzymes, disrupting their structure and function. Additionally, SeNPs impair natural ion and nutrient transport processes across cell walls, preventing essential cellular functions. Furthermore, their inhibitory effects may also be associated with DNA degradation or disruption of enzyme activity owing to the generation of hydroxyl free radicals [29,61].

### 3.3 In Vivo wound healing activity of PSp-SeNPs

**Fig. 4:**
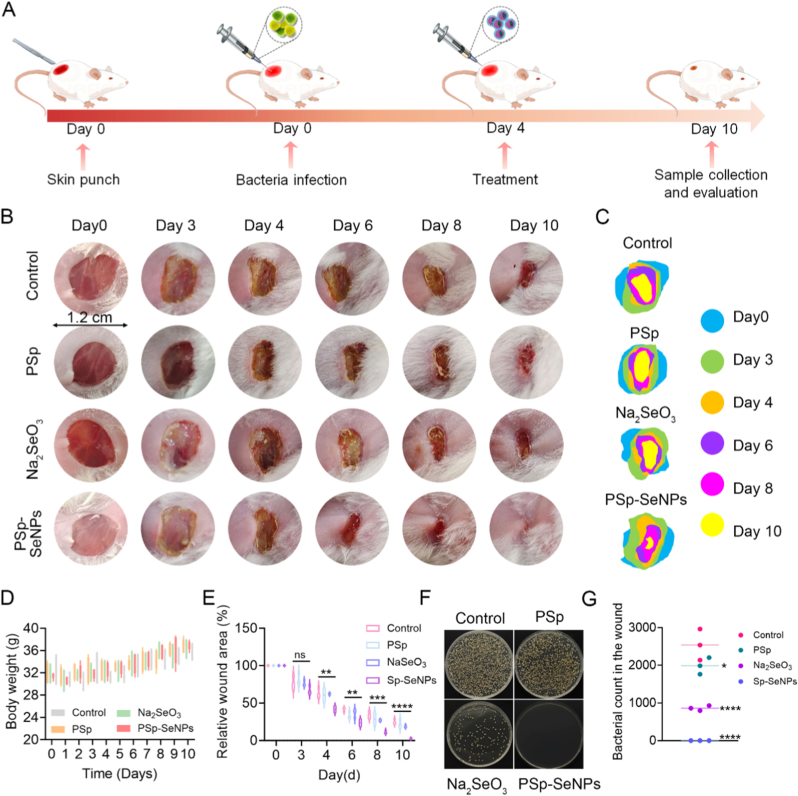
*In Vivo* wound healing activity of PSp-SeNPs. (A) Schematic representative diagram of the wound-healing and antibacterial therapeutic processes. (B) Wound healing progression images of the untreated group (control) and treated groups (PSp, Na_2_SeO_3_, and PSp-SeNPs). Scale bars = 12 mm (1.2 cm). (C) Graphical representation of the wound area in the treated groups at the specified time points. Colors correspond to specific groups and days. (D) Body weight monitoring after treatment administration. (E) Graph displaying the change in the relative wound area percentage of the treatment groups (PSp, Na_2_SeO_3_, and PSp-SeNPs) over time. (F) Bacterial cultures after different treatment groups. (G) Dot plot showing the bacterial count in wound samples after different treatment groups. Quantitatively illustrates the effectiveness of the treatment groups in controlling the bacterial load. Data represent mean ± SD (n = 6), with various significance *∗p < 0.05, ∗∗p < 0.01, ∗∗∗p < 0.001, ∗∗∗∗p < 0.0001*. (For interpretation of the references to color in this figure legend, the reader is referred to the Web version of this article.)

Research has shown that SeNPs promote faster wound healing [21,36,62]. Concurrently, plant-derived natural compounds further enhance healing outcomes by minimizing scar formation and aiding blood clotting [63,64]. Thus, the synergetic potential of natural compound extract-derived SeNPs in wound healing is a promising novel therapeutic approach in nanomedicine [20]. To investigate this, a mouse model with skin-infected wounds was treated with PSp-SeNPs, Na_2_SeO_3_, or PSp (Fig. 4A) to assess their potential in wound healing. Wound closure was monitored and documented photographically (Fig. 4B). Over a 10-day treatment period, no adverse effects occurred. The wound area ratio decreased progressively in the Na_2_SeO_3_ and PSp-SeNPs-treated groups compared with the control and PSp groups, showing accelerated healing, especially at approximately days 6 and 10 (Fig. 4C). PSp-SeNPs treatment resulted in complete wound closure by day 10, with no significant body weight changes observed (Fig. 4D), as evidenced by wound healing rates quantification (Fig. 4E). Bacterial colony analysis revealed high bacterial growth in the control and PSp-treated groups. Conversely, treatment with Na_2_SeO_3_ and the PSp-SeNPs showed a significant decrease in the number of colonies (Fig. 4F), indicating effective infection management. Together, this confirms PSp-SeNPs significantly accelerated wound closure compared to all other treated groups (Fig. 4E) and reduced bacterial load by > 4-fold versus controls (Fig. 4G). These findings align with those documented in earlier studies, where natural compound extract-derived SeNPs demonstrated a significant wound-healing process [36,42]. Furthermore, treatment with SeNPs significantly accelerated wound healing, as evidenced by the reduction in wound diameter after just 11 days [21]. Collectively, the results demonstrated that after ten days, PSp-SeNPs are essential for restoring skin layers, controlling infection, and accelerating wound healing.

Additionally, the pronounced wound closure and >4-fold reduction in bacterial load achieved with PSp-SeNPs (Fig. 4E–G) highlight a significant advantage over some chemically synthesized SeNP systems. Chemically synthesized SeNPs, while effective, often show reduced *in vivo* efficacy due to aggregation, lower bioavailability, and potential toxicity concerns at effective doses [23,25]. The PSp extract coating not only stabilizes the nanoparticles but also contributes bioactive anti-inflammatory and antioxidant effects, creating a multi-mechanistic therapeutic platform that outperforms both its individual components (Na_2_SeO_3_ and PSp) and conventional synthetic counterparts.

### 3.4 Histological and anti-inflammatory activity of PSp-SeNPs

**Fig. 5:**
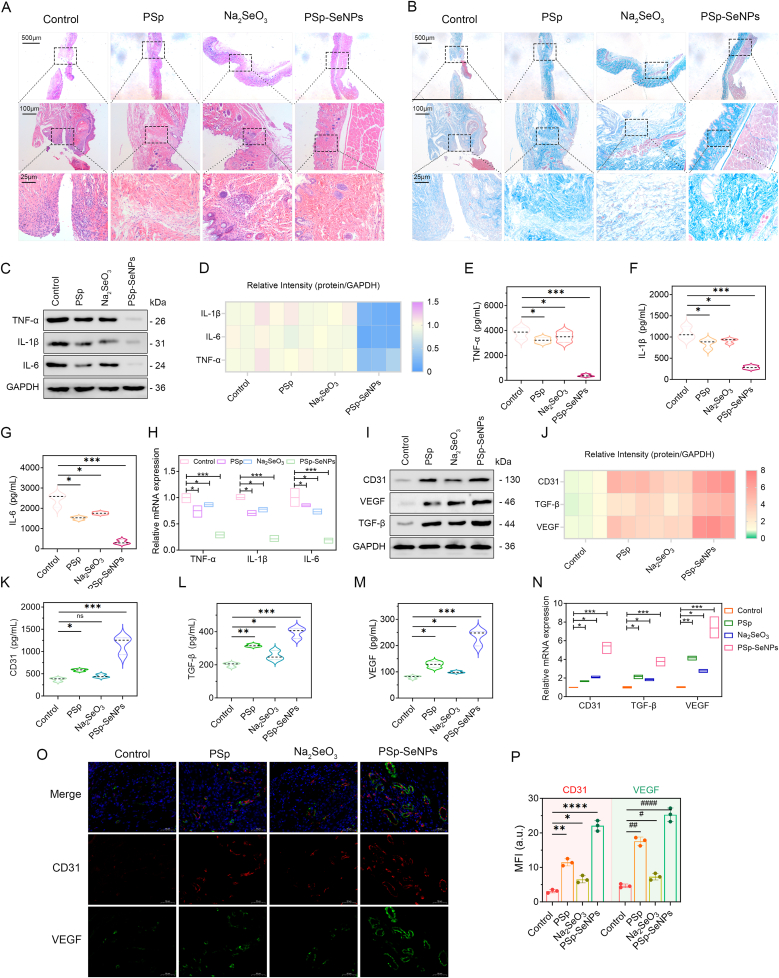
Histological and anti-inflammatory activity of PSp-SeNPs. (A–B) Representative images of H&E-stained histopathological sections (A) and Masson staining (B) of wound tissue on day 10 after treatment, with scale bars of 500 μm, 100 μm, and 25 μm. (C) Protein expression of the cytokines TNF-α, IL-1β, and IL-6 after treatment with PSp, Na_2_SeO_3_, or PSp-SeNPs. (D) Heat map of cytokines TNF-α, IL-1β, and IL-6 protein intensity after treatment. The color bar indicates expression levels ranging from low (blue) to high (pink). (E–G) ELISA quantification of TNF-α (E), IL-1β (F), and IL-6 (G) protein expressions after treatment. (H) qRT‒PCR analysis of TNF-α, IL-1β, and IL-6 after treatments. (I) Protein expression of TGF-β, VEGF, and CD31 following treatment. (J) Heat map of relative TGF-β, VEGF, and CD31 protein intensity. (K–M) ELISA quantifications of TGF-β (K), VEGF (L), and CD31 (M) protein expression levels after treatment. (N) mRNA expression levels of TGF-β, VEGF, and CD31 after treatments. (O, P) Immunofluorescence staining of CD31 and VEGF (O), and (P) quantification of CD31 and VEGF in wound tissue (n = 3). Scale bar = 50 μm. Data represent mean ± SD (n = 3), with different significance *∗p < 0.05, ∗∗p < 0.01, ∗∗∗p < 0.001, ∗∗∗∗p < 0.0001*. (For interpretation of the references to color in this figure legend, the reader is referred to the Web version of this article.)

Histological assessment of wound healing is crucial for understanding the dynamics of healing in patient management [65]. To investigate the therapeutic effect of PSp-SeNPs on wound healing, we conducted a histological analysis. H&E staining of the wound sections showed faster epithelial regeneration and less infiltration of inflammatory cells into the epidermis and dermis 10 days after PSp-SeNPs treatment than after Na_2_SeO_3_ or PSp treatment (Fig. 5A). Furthermore, Masson's trichrome staining unveiled consistently dense and structured subcutaneous collagen fibers at 10-days in the PSp-SeNPs-treated groups in contrast to those in the Na_2_SeO_3_ and PSp-treated groups (Fig. 5B). It has been reported that treatments using chitosan/SeNPs enhance collagen maturation, contributing to wound strength and tissue repair [66]. SeNPs have repeatedly been shown to accelerate wound closure and promote collagen production, fibroblast proliferation, and angiogenesis in various animal models [36,42].

The release of cytokines such as IL-1β, IL-6, and TNF-α during excessive inflammation disrupts the crucial wound repair process, leading to increased fibrosis and scar formation [67,68]. Bacterial infections can further exacerbate this inflammation, impairing the healing process. To gain more insight into this process, we quantified the cytokine levels in the wound sections. The results revealed that the protein expression levels of the cytokines TNF-α, IL-1β, and IL-6 significantly decreased within the wound tissue by 10 days of PSp-SeNPs treatment compared with those of Na_2_SeO_3_ and PSp treatment (Fig. 5C and D). These results were further confirmed by ELISA (Fig. 5E–G), and qRT‒PCR analysis demonstrated a decrease in the mRNA expression of TNF-α, IL-1β, and IL-6 (Fig. 5H). PSp-SeNPs effectively modulated the inflammatory response and accelerated wound repair, consistent with previous research [44].

**Fig. 6:**
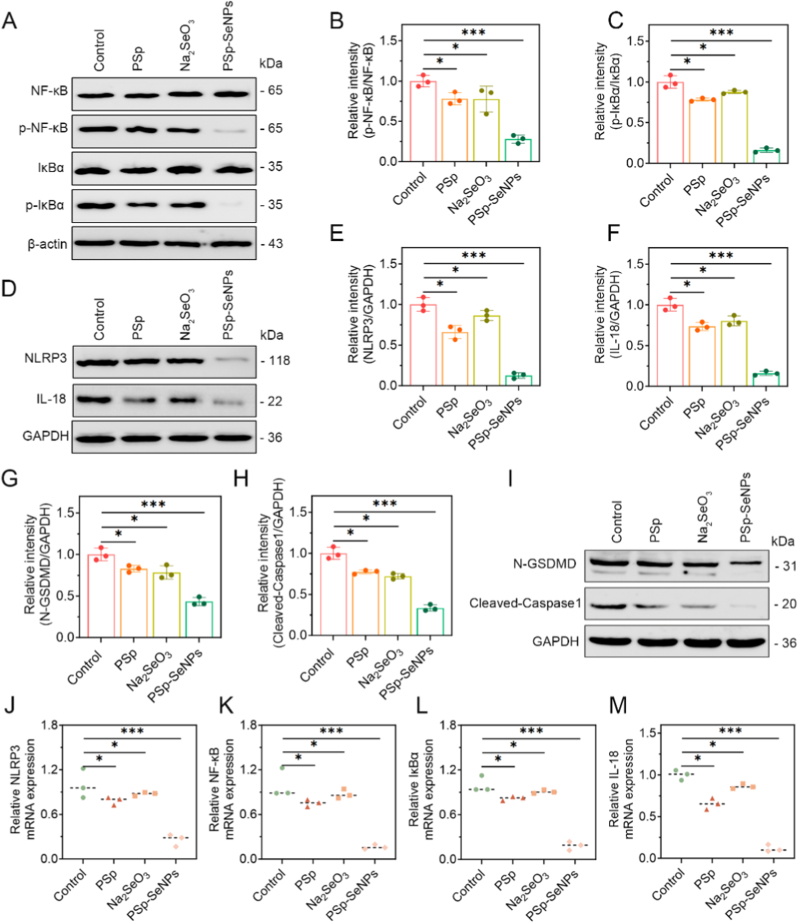
PSp-SeNPs impair pyroptosis to promote wound healing. (A) Western blot analysis of NF-κB, IκBα, and the phosphorylated forms (p-NFκB and p-IkBα) after PSp, Na_2_SeO_3_, and PSp-SeNPs treatments. (B–C) Quantifications of p-NF-κB (B) and p-IκBα (C) protein expressions after treatment with PSp, Na_2_SeO_3_, and PSp-SeNPs. (D) Western Blot analysis of NLRP3 and IL-18 after treatments. (E–F) Quantifications of NLRP3 (E) and IL-18 (F) protein expression after treatments. (G, H) Quantifications of N-GSDMD (G) and cleaved-caspase-1 (H) protein expressions after different treatments. (I) Western Blot analysis of N-GSDMD and cleaved-caspase1 protein expression levels after treatment with PSp, Na_2_SeO_3_, and PSp-SeNPs. (J–M) qRT‒PCR analysis of NLRP3 (J), NF-κB (K), IκBα (L), and pro-IL-18 (M) mRNA expression levels after different treatments. Data represent mean ± SD (n = 3), with different significance *∗p < 0.05, ∗∗p < 0.01, ∗∗∗p < 0.001, ∗∗∗∗p < 0.0001*.

Angiogenesis, fibroblast proliferation, and collagenogenesis are critical for wound healing. SeNPs have been shown to increase the levels of cell adhesion molecule-1 (CD31), vascular endothelial growth factor (VEGF), and transforming growth factor-beta (TGF-β), thereby accelerating wound closure and enhancing the healing outcomes [69,70]. Consistent with this finding, PSp-SeNPs treatment significantly increased TGF-β, VEGF, and CD31 protein levels in wound tissue by 10 days compared to Na_2_SeO_3_ and PSp groups (Fig. 5I and J). The low CD31 expression observed via western blot in the control group (Fig. 5I) reflects *impaired angiogenesis* in untreated infected wounds, consistent with the pathophysiology of delayed healing in *S. aureus*-infected murine models [3,5]. This is not indicative of a complete absence of blood vessels but rather signifies severely compromised angiogenic activity. ELISA (Fig. 5K–M) and qRT‒PCR analysis (Fig. 5N) supported this result, which further aligned with histological findings. Similarly, immunofluorescence staining analysis evaluating angiogenesis markers CD31 and VEGF showed that PSp-SeNPs exhibited the strongest co-localization signal of these markers compared to the control **(**Fig. 5O), suggesting substantial formation of new blood vessels. Quantification analysis further confirmed a significant increase in the CD31/VEGF expression after treatment with PSp-SeNPs **(**Fig. 5P). This vascular remodeling underpins accelerated healing, as new vessels facilitate nutrient delivery and immune cell trafficking. The effect of PSp-SeNPs surpasses Na_2_SeO_3_ or PSp extract alone, underscoring the therapeutic advantage of nano-formulated selenium combined with plant-derived bioactives. Notably, suppressed pyroptosis (Fig. 6) likely contributes to this pro-angiogenic milieu by reducing inflammatory cytokines that antagonize VEGF signaling. Together, these results suggest that PSp-SeNPs may be promising for wound healing and tissue regeneration by both downregulating inflammatory cytokines and upregulating molecular markers associated with angiogenesis, fibroblast proliferation, and collagenesis.

### 3.5 PSp-SeNPs impair pyroptosis to promote wound healing

Pyroptosis is essential to the body's natural wound repair mechanism [71]. However, exacerbated pyroptosis triggered by pathogenic microorganisms promotes the sustained activation of inflammasomes, resulting in the cleavage of gasdermin D (GSDMD) and the release of proinflammatory cytokines such as IL-1β and IL-18 [11,72], which cause inflammatory tissue damage and serious complications as previously reported [[73], [74], [75], [76]]. Thus, regulating pyroptosis may serve as a promising therapeutic strategy in wound healing. Given this evidence, we harnessed the effects of PSp-SeNPs on NLRP3-mediated pyroptosis and its associated markers to promote wound healing. As expected, the level of phosphorylated NF-κB (p-NF-κB) was significantly lower after PSp-SeNPs treatment than after Na_2_SeO_3_ or PSp treatment (Fig. 6A and B), demonstrating the inhibition of NF-κB activation, which is crucial for inflammatory responses. The protein expression levels of p-IκBα/IκBα, IL-1β (Fig. 5C and F), and IL-18 (Fig. 6D and F) were significantly lower after PSp-SeNPs treatment than after Na_2_SeO_3_ or PSp treatment. Concurrently, PSp-SeNPs treatment impaired the protein expression of NLRP3 more than Na_2_SeO_3_ or PSp (Fig. 6D and E).

Furthermore, quantitative analysis revealed that treatments with Psp and Na_2_SeO_3_ moderately reduced expression levels of both the N-terminal fragment of GSDMD (N-GSDMD) and the active Caspase-1 p20 subunit compared to controls. Psp-SeNPs induced the most significant decrease (Fig. 6G and H). Immunoblotting confirmed a substantial reduction in N-GSDMD and cleaved Caspase-1 protein expression following Psp-SeNP treatment (Fig. 6I), indicating impaired pyroptosis-related pathway activation. Caspase-1 (p20) is essential for pyroptosis, cleaving GSDMD within inflammasomes to generate the N-terminal fragment. This fragment oligomerizes, forming membrane pores that lead to cell lysis [77,78]. By inhibiting GSDMD pore formation, Psp-SeNPs may confer protection against cell death, potentially through modulation of inflammasome activity and GSDMD-mediated pyroptosis, key drivers of chronic wound inflammation. Moreover, qRT‒PCR analysis aligned with this result by demonstrating significantly lower mRNA expression levels of NLRP3 inflammasome (Fig. 6J), NF-κB (Fig. 6K), IκBα (Fig. 6L), and IL-18 (Fig. 6M) following treatment with PSp-SeNPs than after Na_2_SeO_3_ or PSp treatment. This result showed that PSP-SeNPs not only suppressed NLRP3 expression but also potently inhibited caspase-1 activation and GSDMD cleavage, the definitive executors of pyroptosis. This dual inhibition at both signaling and execution levels explains their superior anti-inflammatory efficacy in promoting wound healing.

Notably, activation of the canonical NLRP3 inflammasome is crucial in wound healing and requires two signals [71,[79], [80], [81]]. First, NF-κB priming upregulates NLRP3 and precursor interleukin isoforms, such as pro-IL-1β/pro-IL-18. Second, NLRP3 oligomerization triggers caspase-1 activation platforms (ASC inflammasomes), synergically regulating post-injury inflammation and pyroptotic cell death. Consistently, by suppressing the activation of NLRP3-mediated pyroptosis, PSp-SeNPs reduced the downstream inflammatory cascade and accelerated wound healing. Research has shown that SeNPs mitigate liver inflammation and damage from microplastics through the NF-κB/NLRP3 signaling pathway [82]. Additionally, Nano-Se antagonizes BDE-209-induced pyroptotic death in cardiovascular endothelia through endoplasmic reticulum stress-thioredoxin-interacting protein-NLRP3 inflammasome axis [26]. Combined Mulberry leaf and fruit extract (MLFE) has been shown to regulate NLRP3 inflammasome levels by suppressing skin inflammation during the early stages of wound healing in obese individuals [83]. Similarly, Traditional Chinese medicine-based SeNPs are reported to have anti-inflammatory and antimicrobial effects, which may help treat microbial infectious diseases by modulating pyroptosis in host cells [84]. Further research has supported the potential of natural bioactive compounds extracted from PSp in suppressing the NLRP3 inflammasome, thereby preventing excessive pyroptosis [85].

Evidence indicates PSp [[86], [87], [88], [89]] and SeNPs [26,27,82] alleviate inflammation in various diseases by regulating NLRP3-mediated pyroptosis. Nevertheless, research on signaling pathway interactions in wound healing and on naturally sourced SeNPs targeting NLRP3-mediated pyroptosis for wound repair is lacking. Thus, this work offers novel perspectives into the in-depth mechanisms of pyroptosis regulation and inflammasomes in wound healing. Furthermore, the work demonstrates the potential of PSp-SeNPs as innovative therapeutic targets for enhancing wound healing by controlling NLRP3-mediated pyroptosis.

### 3.6 Biocompatibility assessment of PSp-SeNPs *in vivo*

Biocompatibility is crucial for green-synthesized PSp-SeNPs in biomedical applications, as it prevents adverse health effects. Studies have shown that SeNPs from natural sources are biocompatible and exhibit low toxicity in cellular and animal models [25,27,48]. To explore this, we conducted a histological analysis of major organs (heart, liver, spleen, lung, and kidney) after treatment with PSp-SeNPs, Na_2_SeO_3_, and PSp.

**Fig. 7:**
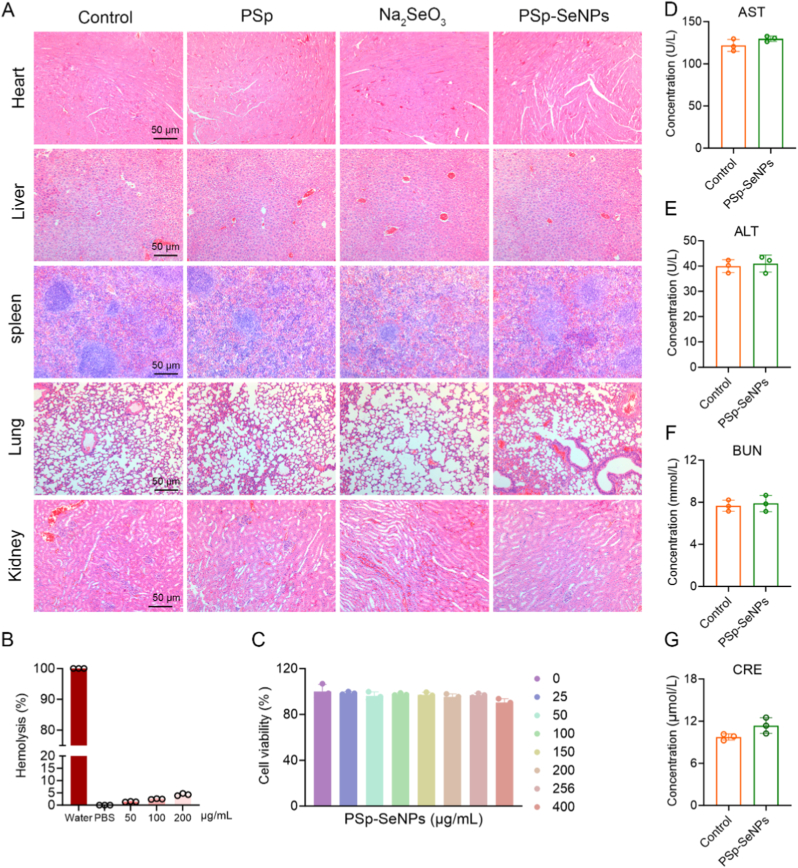
Biocompatibility assessment of PSp-SeNPs. (A) H&E-stained sections of the major organs, heart, liver, spleen, lung, and kidney, after different treatments. Scale bar = 50 μm. (B) Hemolysis analysis of PSp-SeNPs across tested concentrations (n = 3). (C) CCK-8 assay showing the viability of HUVEC cells after treatment with different concentrations of PSp-SeNPs (25–400 μg/mL) for 24 h. (D–G) Blood biochemistry analysis of AST, ALT, CRE, and BUN in control and PSp-SeNPs-treated mice (n = 3).

H&E staining revealed significantly improved tissue morphology, characterized by low inflammatory cell infiltration and better organization after treatment with PSp-SeNPs relative to Na_2_SeO_3_ or PSp (Fig. 7A). This suggests PSp-SeNPs are well-tolerated and promote tissue health, supporting their potential therapeutic safety applications. These findings are consistent with previous research showing that plant-derived SeNPs are superior to elemental selenium in biocompatibility [19], and that the green-synthesized SeNPs from *Allium sativum* are less toxic than chemically synthesized SeNPs [90]. Similarly, PF-SeNPs are less toxic than chemically synthesized sodium selenite (Na_2_SeO_3_) [51]. Overall, PSp-SeNPs enhanced biocompatibility and bioavailability without adverse effects, supporting their potential for therapeutic application. *However, it is crucial to acknowledge that the acute murine wound model does not fully emulate the complexity of chronic human wounds, which involve persistent inflammation, metabolic dysregulation associated with diabetes, and biofilm formation. Future studies could validate PSp-SeNPs in chronic diabetic wounds, biofilm-infected, and aged animal models to assess translational relevance.*

Moreover, standard *in vitro* hemolysis assays confirmed the blood compatibility of *PSp-SeNPs*, showing hemolysis rates under 5 % at all concentrations investigated (Fig. 7B), consistent with previous research [91,92]. Similarly, PSp-SeNPs likewise showed minimal erythrocyte damage, maintaining excellent hemocompatibility even at 200 μg/mL. Potential systemic effects of PSp-SeNPs were further assessed by measuring serum markers for liver (AST and ALT) and kidney (BUN and CRE) function 24 h post-administration, supported by histopathological analysis. Serum concentrations of these critical biomarkers, recognized indicators of drug-induced injury [93], were within normal ranges in treated mice (Fig. 7C–F), demonstrating no detectable harm to liver or kidney function. Collectively, the biocompatibility of green-synthesized SeNPs is a critical advantage over their chemically synthesized analogues. Chemically synthesized SeNPs, often stabilized with synthetic ligands, have been frequently associated with dose-dependent cytotoxicity in mammalian cells and hemolytic activity [23,25]. In contrast, our PSp-SeNPs demonstrated excellent hemocompatibility (<5 % hemolysis at 200 μg/mL, Fig. 7B) and no observed systemic toxicity (Fig. 7C–G, 7A), a finding consistent with other plant-derived SeNPs [25,51]. This superior safety profile is directly linked to the natural biomolecules from the PSp extract that cap and stabilize the SeNPs, mitigating the reactive surface characteristics that often cause toxicity in chemically synthesized SeNPs.

## 4 Conclusion

This work demonstrates PSp-SeNPs' encouraging potential as potent wound healing therapeutic agents, offering a superior alternative to traditional chemically synthesized SeNPs in terms of antibacterial efficacy, multi-mechanistic healing promotion via pyroptosis regulation, and exceptional biocompatibility. PSp-SeNPs enhanced antibacterial activity against significant clinical pathogens such as *S. aureus* and MRSA, which aids infection prevention in wound care. Furthermore, PSp-SeNPs regulated pyroptosis, a unique mechanism that modulates inflammation, promoting repair and regeneration of cells and tissues. PSp-SeNPs demonstrated excellent biocompatibility, evidenced by histopathological organ integrity, hemocompatibility (<5 % hemolysis), absence of systemic immune activation, and normal hepatic/renal biomarkers, suggesting considerable therapeutic promise for broader biomedical applications. Beyond topical wound management, the strong antibiofilm properties and biocompatibility of PSp-SeNPs provide a promising application in implantable medical devices, such as coatings to prevent microbial colonization on prosthetics or catheters. They could also be implantable as antimicrobial coatings for surgical instruments to reduce biofilm-associated infections. The prospects of green-synthesized SeNPs, such as PSp-SeNPs, represent a potential breakthrough in addressing the complexities of wound healing and other biomedical challenges. While effective in acute murine wounds, thorough clinical trials are essential to confirm their efficacy and practical value.

## CRediT authorship contribution statement

**Chen Chen:** Writing – original draft, Methodology, Investigation, Conceptualization. **Fructueux Modeste Amona:** Investigation, Formal analysis. **Ziqi Sha:** Validation, Software. **Jiamin Li:** Methodology. **Yongding Ke:** Data curation. **Yuxin You:** Supervision, Resources. **Luyuan Yang:** Resources. **Guangfu Liao:** Writing – review & editing, Supervision, Methodology, Conceptualization. **Xi Chen:** Writing – review & editing, Supervision, Funding acquisition. **Yipeng Pang:** Writing – review & editing, Project administration, Funding acquisition, Conceptualization. **Yi Liu:** Writing – review & editing, Supervision, Funding acquisition.

## Declaration of competing interest

The authors declare that they have no known competing financial interests or personal relationships that could have appeared to influence the work reported in this paper.

## Data Availability

Data will be made available on request.

## References

[1] Li T., Sun W., Qian D., Wang P., Liu X., He C., Chang T., Liao G., Zhang J. (2025). Plant-derived biomass-based hydrogels for biomedical applications. Trends Biotechnol..

[2] Jia R., He C., Wang S., Gao Y., Song L., Wang P., Liao G., Shi X. (2024). Recent advances in graphitic carbon nitride-based heterojunction for biomedical applications. Chem. Eng. J..

[3] Bessa L.J., Fazii P., Di Giulio M., Cellini L. (2015). Bacterial isolates from infected wounds and their antibiotic susceptibility pattern: Some remarks about wound infection. Int. Wound J..

[4] Wang F., Fan Y., Liu Y., Lou X., Sutrisno L., Peng S., Li J. (2024). Oxygen-carrying semiconducting polymer nanoprodrugs induce sono-pyroptosis for deep-tissue tumor treatment. Exploration.

[5] Martin P., Pardo-Pastor C., Jenkins R.G., Rosenblatt J. (2024). Imperfect wound healing sets the stage for chronic diseases. Science.

[6] Cefalu J.E., Barrier K.M., Davis A.H. (2017). Wound infections in critical care. Crit. Care Nurs. Clin..

[7] Spichler A., Hurwitz B.L., Armstrong D.G., Lipsky B.A. (2015). Microbiology of diabetic foot infections: from Louis Pasteur to “crime scene investigation,”. BMC Med..

[8] Lipsky B.A., Berendt A.R., Cornia P.B., Pile J.C., Peters E.J.G., Armstrong D.G., Deery H.G., Embil J.M., Joseph W.S., Karchmer A.W., Pinzur M.S., Senneville E. (2012). Infectious diseases society of America clinical practice guideline for the diagnosis and treatment of diabetic foot infections. Clin. Infect. Dis..

[9] Dubyak G.R., Miller B.A., Pearlman E. (2023). Pyroptosis in neutrophils: multimodal integration of inflammasome and regulated cell death signaling pathways. Immunol. Rev..

[10] Bergsbaken T., Fink S.L., Cookson B.T. (2009). Pyroptosis: Host cell death and inflammation. Nat. Rev. Microbiol..

[11] Brokatzky D., Mostowy S. (2022). Pyroptosis in host defence against bacterial infection. DMM Disease Models and Mechanisms.

[12] Lv D., Cao X., Zhong L., Dong Y., Xu Z., Rong Y., Xu H., Wang Z., Yang H., Yin R., Chen M., Ke C., Hu Z., Deng W., Tang B. (2023). Targeting phenylpyruvate restrains excessive NLRP3 inflammasome activation and pathological inflammation in diabetic wound healing. Cell Rep. Med..

[13] Zhao K., An R., Xiang Q., Li G., Wang K., Song Y., Liao Z., Li S., Hua W., Feng X., Wu X., Zhang Y., Das A., Yang C. (2021). Acid-sensing ion channels regulate nucleus pulposus cell inflammation and pyroptosis via the NLRP3 inflammasome in intervertebral disc degeneration. Cell Prolif..

[14] Wan L., Bai X., Zhou Q., Chen C., Wang H., Liu T., Xue J., Wei C., Xie L. (2022). The advanced glycation end-products (AGEs)/ROS/NLRP3 inflammasome axis contributes to delayed diabetic corneal wound healing and nerve regeneration. Int. J. Biol. Sci..

[15] Ding Y., Ding X., Zhang H., Li S., Yang P., Tan Q. (2022). Relevance of NLRP3 inflammasome-related pathways in the pathology of diabetic wound healing and possible therapeutic targets. Oxid. Med. Cell. Longev..

[16] Mirza R.E., Fang M.M., Weinheimer-Haus E.M., Ennis W.J., Koh T.J. (2014). Sustained inflammasome activity in macrophages impairs wound healing in type 2 diabetic humans and mice. Diabetes.

[17] Thakkar K.N., Mhatre S.S., Parikh R.Y. (2010). Biological synthesis of metallic nanoparticles. Nanomedicine.

[18] Chen Z., Yan D., Wang X., Ding G., Wang Z., Xiao Y., Liu X., Wang P., Chen L., Shuai L., Liao G. (2025). Biochar-tailored carbon nitride enables Piezo-Photocatalytic H_2_O_2_ production via boosted charge transport. ACS Catal..

[19] Zhang Y., Dai L., Yang C., Wei B., Liao G. (2025). Spider web-inspired gelatin-based bioplastic enables closed-loop recyclable, biodegradable, and sustainable packaging. Green Chem..

[20] Liao G., Zhang L., Li C., Liu S.-Y., Fang B., Yang H. (2022). Emerging carbon-supported single-atom catalysts for biomedical applications. Matter.

[21] Nag S., Kar S., Mishra S., Stany B., Seelan A., Mohanto S., Haryini S S., Kamaraj C., Subramaniyan V. (2024). Unveiling green synthesis and biomedical Theranostic paradigms of selenium nanoparticles (SeNPs) - A state-of-the-art comprehensive update. Int. J. Pharm..

[22] Chen C., Wang Y., Zhang D., Wu X., Zhao Y., Shang L., Ren J., Zhao Y. (2021). Natural polysaccharide based complex drug delivery system from microfluidic electrospray for wound healing. Appl. Mater. Today.

[23] Sampath S., Sunderam V., Manjusha M., Dlamini Z., Lawrance A.V. (2024). Selenium nanoparticles: A comprehensive examination of synthesis techniques and their diverse applications in medical research and toxicology studies. Molecules.

[24] Fang M., Zhang H., Wang Y., Zhang H., Zhang D., Xu P. (2023). Biomimetic selenium nanosystems for infectious wound healing. Eng. Regen..

[25] Hashem A.H., Salem S.S. (2022). Green and ecofriendly biosynthesis of selenium nanoparticles using Urtica dioica (stinging nettle) leaf extract: Antimicrobial and anticancer activity. Biotechnol. J..

[26] Jiang Y., Dong B., Jiao X., Shan J., Fang C., Zhang K., Li D., Xu C., Zhang Z. (2024). Nano-selenium alleviates the pyroptosis of cardiovascular endothelial cells in chicken induced by decabromodiphenyl ether through ERS-TXNIP-NLRP3 pathway. Sci. Total Environ..

[27] Liu S., Chen Q., Yan L., Ren Y., Fan J., Zhang X., Zhu S. (2022). Phytosomal tripterine with selenium modification attenuates the cytotoxicity and restrains the inflammatory evolution via inhibiting NLRP3 inflammasome activation and pyroptosis. Int. Immunopharmacol..

[28] Pyrzynska K., Sentkowska A. (2022). Biosynthesis of selenium nanoparticles using plant extracts. J. Nanostruct. Chem..

[29] Sentkowska A., Konarska J., Szmytke J., Grudniak A. (2024). Herbal polyphenols as selenium reducers in the green synthesis of selenium nanoparticles: antibacterial and antioxidant capabilities of the obtained SeNPs. Molecules.

[30] Li A., Xiao R., He S., An X., He Y., Wang C., Yin S., Wang B., Shi X., He J. (2019). Research advances of purple sweet potato anthocyanins: Extraction, identification, stability, bioactivity, application, and biotransformation. Molecules.

[31] Yun D., Wu Y., Yong H., Tang C., Chen D., Kan J., Liu J. (2024). Recent advances in purple sweet potato anthocyanins: Extraction, isolation, functional properties and applications in biopolymer-based smart packaging. Foods.

[32] Teow C.C., Den Truong V., McFeeters R.F., Thompson R.L., Pecota K.V., Yencho G.C. (2007). Antioxidant activities, phenolic and β-carotene contents of sweet potato genotypes with varying flesh colours. Food Chem..

[33] Liao G., Sun E., Kana E.B.G., Huang H., Sanusi I.A., Qu P., Jin H., Liu J., Shuai L. (2024). Renewable hemicellulose-based materials for value-added applications. Carbohydr. Polym..

[34] Garza-García J.J.O., Hernández-Díaz J.A., León-Morales J.M., Velázquez-Juárez G., Zamudio-Ojeda A., Arratia-Quijada J., Reyes-Maldonado O.K., López-Velázquez J.C., García-Morales S. (2023). Selenium nanoparticles based on Amphipterygium glaucum extract with antibacterial, antioxidant, and plant biostimulant properties. J. Nanobiotechnol..

[35] Chandramohan S., Sundar K., Muthukumaran A. (2019). Hollow selenium nanoparticles from potato extract and investigation of its biological properties and developmental toxicity in zebrafish embryos. IET Nanobiotechnol..

[36] Karas R.A., Alexeree S., Elsayed H., Attia Y.A. (2024). Assessment of wound healing activity in diabetic mice treated with a novel therapeutic combination of selenium nanoparticles and platelets rich plasma. Sci. Rep..

[37] Balouiri M., Sadiki M., Ibnsouda S.K. (2016). Methods for in vitro evaluating antimicrobial activity: A review. J. Pharm. Anal..

[38] Lewis J.S., Mathers Amy J., Bobenchik April M., Lynn Bryson Alexandra, Campeau Shelley, Cullen Sharon K., Dingle Tanis, Galas Marcelo F., Humphries Romney M., Kirn Thomas J. (2024). https://clsi.org/media/pjfbviql/m100ed34e_sample.pdf.

[39] Shakibaie M., Forootanfar H., Golkari Y., Mohammadi-Khorsand T., Shakibaie M.R. (2015). Anti-biofilm activity of biogenic selenium nanoparticles and selenium dioxide against clinical isolates of Staphylococcus aureus, Pseudomonas aeruginosa, and Proteus mirabilis. J. Trace Elem. Med. Biol..

[40] Mempin R., Tran H., Chen C., Gong H., Kim Ho K., Lu S. (2013). Release of extracellular ATP by bacteria during growth. BMC Microbiol..

[41] Dai T., Kharkwal G.B., Tanaka M., Huang Y.Y., Bil de Arce V.J., Hamblin M.R. (2011). Animal models of external traumatic wound infections. Virulence.

[42] Golmohammadi R., Najar-Peerayeh S., Tohidi Moghadam T., Hosseini S.M.J. (2020). Synergistic antibacterial activity and wound healing properties of selenium-chitosan-mupirocin Nanohybrid system: an in vivo study on Rat diabetic Staphylococcus aureus wound infection model. Sci. Rep..

[43] Standage S.W., Caldwell C.C., Zingarelli B., Wong H.R. (2012). Reduced peroxisome proliferator-activated receptor α expression is associated with decreased survival and increased tissue bacterial load in sepsis. Shock.

[44] El-Sayed H., Morad M.Y., Sonbol H., Hammam O.A., Abd El-Hameed R.M., Ellethy R.A., Ibrahim A.M., Hamada M.A. (2023). Myco-synthesized selenium nanoparticles as wound healing and antibacterial agent: An in vitro and in vivo investigation. Microorganisms.

[45] Suhendy H., Fidrianny I., Insanu M. (2023). Phytochemical compounds and pharmacological activities of Ipomoea batatas L.: An updated review. Pharmacia.

[46] Ghasemzadeh A., Talei D., Jaafar H.Z.E., Juraimi A.S., Mohamed M.T.M., Puteh A., Halim M.R.A. (2016). Plant-growth regulators alter phytochemical constituents and pharmaceutical quality in Sweet potato (Ipomoea batatas L.). BMC Compl. Alternative Med..

[47] Xiao Y., Ding G., Tao J., Wang Z., Chen Z., Chen L., Shuai L., Liao G. (2025). Selective conversion of CO_2_ to C_2_H_6_ in pure water photocatalyzed by fluorobenzene-linked perylene diimide. Nat. Commun..

[48] Liang S., Liu Y., Zhu H., Liao G., Zhu W., Zhang L. (2024). Emerging nitric oxide gas-assisted cancer photothermal treatment. Exploration.

[49] Ge Y.M., Xue Y., Zhao X.F., Liu J.Z., Xing W.C., Hu S.W., Gao H.M. (2024). Antibacterial and antioxidant activities of a novel biosynthesized selenium nanoparticles using Rosa roxburghii extract and chitosan: Preparation, characterization, properties, and mechanisms. Int. J. Biol. Macromol..

[50] Huang X., Chen X., Chen Q., Yu Q., Sun D., Liu J. (2016). Investigation of functional selenium nanoparticles as potent antimicrobial agents against superbugs. Acta Biomater..

[51] Gunti L., Dass R.S., Kalagatur N.K. (2019). Phytofabrication of selenium nanoparticles from emblica officinalis fruit extract and exploring its biopotential applications: Antioxidant, antimicrobial, and biocompatibility. Front. Microbiol..

[52] Bužková A., Hochvaldová L., Večeřová R., Malina T., Petr M., Kašlík J., Kvítek L., Kolář M., Panáček A., Prucek R. (2025). Selenium nanoparticles: influence of reducing agents on particle stability and antibacterial activity at biogenic concentrations. Nanoscale.

[53] Hernández-Díaz J.A., Garza-García JoJ.O., León-Morales J.M., Zamudio-Ojeda A., Arratia-Quijada J., Velázquez-Juárez G., López-Velázquez J.C., García-Morales S. (2021). Antibacterial activity of biosynthesized selenium nanoparticles using extracts of calendula officinalis against potentially clinical bacterial strains. Molecules.

[54] Stewart Philip S., Costerton J William (2001). Antibiotic resistance of bacteria in biofilms. Lancet.

[55] Menon S., Agarwal H., Rajeshkumar S., Jacquline Rosy P., Shanmugam V.K. (2020). Investigating the antimicrobial activities of the biosynthesized selenium nanoparticles and its statistical analysis. BioNanoScience.

[56] Khiralla G.M., El-Deeb B.A. (2015). Antimicrobial and antibiofilm effects of selenium nanoparticles on some foodborne pathogens. Lebensm. Wiss. Technol..

[57] Mu X., Evans T.D., Zhang F. (2024). ATP biosensor reveals microbial energetic dynamics and facilitates bioproduction. Nat. Commun..

[58] Huang T., Holden J.A., Heath D.E., O'Brien-Simpson N.M., O'Connor A.J. (2019). Engineering highly effective antimicrobial selenium nanoparticles through control of particle size. Nanoscale.

[59] Jeong Y., Lim D.W., Choi J. (2014). Assessment of size-dependent antimicrobial and cytotoxic properties of silver nanoparticles. Adv. Mater. Sci. Eng..

[60] Serov D.A., Khabatova V.V., Vodeneev V., Li R., Gudkov S.V. (2023). A review of the antibacterial, fungicidal and antiviral properties of selenium nanoparticles. Materials.

[61] Zhang T., Qi M., Wu Q., Xiang P., Tang D., Li Q. (2023). Recent research progress on the synthesis and biological effects of selenium nanoparticles. Front. Nutr..

[62] Wang Z., Zhang P., Ding X., Wang J., Sun Y., Yin C., Wang W., Fan C., Sun D. (2021). Co-delivery of ampicillin and β-lactamase inhibitor by selenium nanocomposite to achieve synergistic anti-infective efficiency through overcoming multidrug resistance. Chem. Eng. J..

[63] Vitale S., Colanero S., Placidi M., Di Emidio G., Tatone C., Amicarelli F., D'Alessandro A.M. (2022). Phytochemistry and biological activity of medicinal plants in wound healing: an overview of current research. Molecules.

[64] Criollo-Mendoza M.S., Contreras-Angulo L.A., Leyva-López N., Gutiérrez-Grijalva E.P., Jiménez-Ortega L.A., Heredia J.B. (2023). Wound healing properties of natural products: mechanisms of action. Molecules.

[65] Van De Vyver M., Boodhoo K., Frazier T., Hamel K., Kopcewicz M., Levi B., Maartens M., Machcinska S., Nunez J., Pagani C., Rogers E., Walendzik K., Wisniewska J., Gawronska-Kozak B., Gimble J.M. (2021). Histology scoring system for murine cutaneous wounds. Stem Cell. Dev..

[66] Abbaszadeh A., Tehmasebi-Foolad A., Rajabzadeh A., Beigi-Brojeni N., Zarei L. (2019). Effects of chitosan/nano selenium biofilm on infected wound healing in rats; an experimental study. Bull Emerg Trauma.

[67] Eming S.A., Krieg T., Davidson J.M. (2007). Inflammation in wound repair: molecular and cellular mechanisms. J. Invest. Dermatol..

[68] Lopes F.B., Sarandy M.M., Novaes R.D., Valacchi G., Gonçalves R.V. (2024). OxInflammatory responses in the wound healing process: a systematic review. Antioxidants.

[69] Zarharan H., Bagherian M., Shah Rokhi A., Ramezani Bajgiran R., Yousefi E., Heravian P., Niazi Khazrabig M., Es-haghi A., Taghavizadeh Yazdi M.E. (2023). The anti-angiogenesis and antioxidant activity of chitosan-mediated synthesized selenium-gold nanostructure. Arab. J. Chem..

[70] Pérez-Díaz M.A., Prado-Prone G., Díaz-Ballesteros A., González-Torres M., Silva-Bermudez P., Sánchez-Sánchez R. (2023). Nanoparticle and nanomaterial involvement during the wound healing process: an update in the field. J. Nanoparticle Res..

[71] Ito H., Kanbe A., Sakai H., Seishima M. (2018). Activation of NLRP3 signalling accelerates skin wound healing. Exp. Dermatol..

[72] Li L., Dickinson M.S., Coers J., Miao E.A. (2023). Pyroptosis in defense against intracellular bacteria. Semin. Immunol..

[73] Deng W., Bai Y., Deng F., Pan Y., Mei S., Zheng Z., Min R., Wu Z., Li W., Miao R., Zhang Z., Kupper T.S., Lieberman J., Liu X. (2022). Streptococcal pyrogenic exotoxin B cleaves GSDMA and triggers pyroptosis. Nature.

[74] Xu D., Wu X., Peng L., Chen T., Huang Q., Wang Y., Ye C., Peng Y., Hu D.L., Fang R. (2021). The critical role of nlrp6 inflammasome in streptococcus pneumoniae infection in vitro and in vivo. Int. J. Mol. Sci..

[75] Wang G., Zhang C., Jiang F., Zhao M., Xie S., Liu X. (2022). NOD2-RIP2 signaling alleviates microglial ROS damage and pyroptosis via ULK1-mediated autophagy during Streptococcus pneumonia infection. Neurosci. Lett..

[76] Gou X., Xu W., Liu Y., Peng Y., Xu W., Yin Y., Zhang X. (2022). IL-6 prevents lung macrophage death and lung inflammation injury by inhibiting GSDME- and GSDMD-mediated pyroptosis during pneumococcal pneumosepsis. Microbiol. Spectr..

[77] Yu P., Zhang X., Liu N., Tang L., Peng C., Chen X. (2021). Pyroptosis: mechanisms and diseases. Signal Transduct. Targeted Ther..

[78] Boucher D., Monteleone M., Coll R.C., Chen K.W., Ross C.M., Teo J.L., Gomez G.A., Holley C.L., Bierschenk D., Stacey K.J., Yap A.S., Bezbradica J.S., Schroder K. (2018). Caspase-1 self-cleavage is an intrinsic mechanism to terminate inflammasome activity. J. Exp. Med..

[79] Mu X., Wu X., He W., Liu Y., Wu F., Nie X. (2022). Pyroptosis and inflammasomes in diabetic wound healing. Front. Endocrinol..

[80] Vasudevan S.O., Behl B., Rathinam V.A. (2023). Pyroptosis-induced inflammation and tissue damage. Semin. Immunol..

[81] Al Mamun A., Shao C., Geng P., Wang S., Xiao J. (2024). The mechanism of pyroptosis and its application prospect in diabetic wound healing. J. Inflamm. Res..

[82] Shen Q., Liu Y., Li J., Zhou D. (2024). Nano-selenium modulates NF-κB/NLRP3 pathway and mitochondrial dynamics to attenuate microplastic-induced liver injury. Nutrients.

[83] Eo H., Lim Y. (2016). Combined mulberry leaf and fruit extract improved early stage of cutaneous wound healing in high-fat diet-induced obese mice. J. Med. Food.

[84] Rao S., Lin Y., Lin R., Liu J., Wang H., Hu W., Chen B., Chen T. (2022). Traditional Chinese medicine active ingredients-based selenium nanoparticles regulate antioxidant selenoproteins for spinal cord injury treatment. J. Nanobiotechnol..

[85] Dong G., Xu N., Wang M., Zhao Y., Jiang F., Bu H., Liu J., Yuan B., Li R. (2021). Anthocyanin extract from purple sweet potato exacerbate mitophagy to ameliorate pyroptosis in klebsiella pneumoniae infection. Int. J. Mol. Sci..

[86] Wang X., Zhang Z.F., Zheng G.H., Wang A.M., Sun C.H., Qin S.P., Zhuang J., Lu J., Ma D.F., Zheng Y.L. (2017). The inhibitory effects of purple sweet potato color on hepatic inflammation is associated with restoration of nad+ levels and attenuation of nlrp3 inflammasome activation in high-fat-diet-treated mice. Molecules.

[87] Tang C., Han J., Chen D., Zong S., Liu J., Kan J., Qian C., Jin C. (2023). Recent advances on the biological activities of purple sweet potato anthocyanins. Food Biosci..

[88] Shan Q., Zheng Y., Lu J., Zhang Z., Wu D., Fan S., Hu B., Cai X., Cai H., Liu P., Liu F. (2014). Purple sweet potato color ameliorates kidney damage via inhibiting oxidative stress mediated NLRP3 inflammasome activation in high fat diet mice. Food Chem. Toxicol..

[89] Sun C., Diao Q., Lu J., Zhang Z., Wu D., Wang X., Xie J., Zheng G., Shan Q., Fan S., Hu B., Zheng Y. (2019). Purple sweet potato color attenuated NLRP3 inflammasome by inducing autophagy to delay endothelial senescence. J. Cell. Physiol..

[90] Anu K., Singaravelu G., Murugan K., Benelli G. (2017). Green-synthesis of selenium nanoparticles using garlic cloves (Allium sativum): biophysical characterization and cytotoxicity on vero cells. J. Cluster Sci..

[91] Gu J., Zhang P., Li H., Wang Y., Huang Y., Fan L., Ma X., Qian X., Xi J. (2024). Cerium-luteolin nanocomplexes in managing inflammation-related diseases by antioxidant and immunoregulation. ACS Nano.

[92] Xu Y., Luo Y., Weng Z., Xu H., Zhang W., Li Q., Liu H., Liu L., Wang Y., Liu X., Liao L., Wang X. (2023). Microenvironment-responsive metal-phenolic nanozyme release platform with antibacterial, ROS scavenging, and osteogenesis for periodontitis. ACS Nano.

[93] Li F., Qiu Y., Xia F., Sun H., Liao H., Xie A., Lee J., Lin P., Wei M., Shao Y., Yang B., Weng Q., Ling D. (2020). Dual detoxification and inflammatory regulation by ceria nanozymes for drug-induced liver injury therapy. Nano Today.

