# Supplementary material for: Green synthesis of purple sweet potato-derived selenium nanoparticles accelerates wound healing through pyroptosis regulation
Source: Mater Today Bio. 2025 Sep 2;35:102269. doi: 10.1016/j.mtbio.2025.102269 (PMC12859606; doi:10.1016/j.mtbio.2025.102269)
Supplement: Multimedia component 1 [file mmc1.docx]

**Supporting Information**

*for*

**Green synthesis of purple *sweet potato*-derived selenium nanoparticles accelerates wound healing through pyroptosis regulation**

Chen Chen^a^, Fructueux Modeste Amona^b^, Ziqi Sha^c^, Jiamin Li^c^, Yongding Ke^b^, Yuxin You^c^, Luyuan Yang^c^, Guangfu Liao^d,^ *, Xi Chen^b,^ *, Yipeng Pang^b, c^ *, and Yi Liu^c,^ *

^a^ College of Hydraulic Engineering, Jiangsu Vocational Institute of Architectural Technology, Xuzhou, 221000, Jiangsu, China;

^b^ Institute of Cellular and Molecular Biology, School of Life Science, Jiangsu Normal University, Xuzhou, 221116, Jiangsu, China;

^c^ Department of Biophysics, School of Life Sciences, Xuzhou Medical University, Xuzhou, 221004, Jiangsu, China.

^d^ College of Material Engineering, Fujian Agriculture and Forestry University, Fuzhou 350002, China.

*Corresponding authors. E-mail addresses: liaogf@mail2.sysu.edu.cn (G. Liao); cxvirus@126.com (X. Chen); pangyipeng@jsnu.edu.cn (Y. Pang); cbpeliuyinew@163.com (Y. Liu)

# Experimental section

## Reagents and bacterial strains

Sodium selenite (Na_2_SeO_3_) was purchased from Adamas in Shanghai (10641C, China), while *sweet potato* extract was obtained from Hansuyuan (Shaanxi, China). Proinflammatory cytokine ELISA kits for IL-1β, IL-6, and TNF-α were purchased from Elabscience Biotechnology (Wuhan, China). Antibodies against IL-1β (16806-1-AP), IL-18 (10663-1-AP), NF-κB (10745-1-AP), phospho-NF-κB (82335-1-RR), IκBα (10268-1-AP), phospho-IκB alpha (Ser32/36) (82349-1-RR), NLRP3 (30109-1-AP), VEGF (81323-2-RR), CD31 (11265-1-AP), Caspase-1 p20 (22915-1-AP) and TGF-β (81746-2-RR) were obtained from Proteintech (Wuhan, China). In contrast, antibodies against IL-6 (TD6087), TNF-α (PY19810), N-GSDMD (pu224937) and β-actin (T40104) were sourced from Abmart (Shanghai, China). Other general analysis-grade chemicals were obtained from Aladdin in China and did not require further purification.

*Staphylococcus aureus* (*S. aureus*, ATCC 25923) and methicillin-resistant *S. aureus* (MRSA, ATCC 43300) strains were obtained from Fuxiang (Shanghai, China).

## Characterization

The synthesized PSp-SeNPs were characterized to evaluate their size, morphology, chemical composition, and stability. Scanning electron microscopy (SEM) (Zeiss Sigma 300) was used to observe the surface morphology and size distribution of the nanoparticles. In contrast, transmission electron microscopy (TEM) (JEOL F200) provided high-resolution images of their internal structure. The elemental composition and chemical states of the Sp-SeNPs were analyzed via X-ray photoelectron spectroscopy (XPS) (Thermo Fisher Scientific, K-Alpha). X-ray diffraction (XRD) analysis (D8 Advance, Bruker) was performed to identify the crystalline structure. Ultraviolet‒visible (UV‒Vis) spectroscopy (Thermo Fisher Scientific, Evolution One) was used to monitor the spectrograms. Dynamic light scattering (DLS) was used to assess the hydrodynamic diameter and size distribution of the nanoparticles in suspension. Zeta potential was measured with a ZetaSizer (Malvern, ZetaSizer Nano ZS) to evaluate the surface charge of the nanoparticles.

## Stability of PSp-SeNPs in different solvents

Stability was assessed by dispersing PSp-SeNPs in deionized water, 0.9% NaCl, PBS, and DMEM at a concentration of 0.5 mg/mL, followed by sonication for 15 minutes. The particle size was monitored over 12 hours via DLS to identify any aggregation or sedimentation. The long-term stability was assessed by storing the dispersions at room temperature and 4°C.

## Histopathological analysis

Histopathology is crucial for monitoring healing progress, evaluating morphological changes, and diagnosing and treating impaired wounds [49]. Wound tissues were fixed in 4% paraformaldehyde and embedded in paraffin wax. The tissue paraffin blocks were cut to a thickness of 4 µm using slide microtomes. The slices were deparaffinized and stained with hematoxylin & eosin (H&E) and Masson's trichrome to observe morphological changes in the wound tissues under a microscope (Leica, Germany) as previously described [26,50,51]. H&E staining of the heart, liver, spleen, lung, and kidney was performed using the same procedure outlined above to assess safety.

## Immunofluorescence staining analysis

Mouse wound tissue sections were air-dried and fixed in 4% paraformaldehyde (PFA) for 10 min at room temperature (RT). After three 5-minute PBS rinses, blocking was conducted using 5% goat serum in PBS for 1 h. Primary antibodies specific for CD31 and VEGF (diluted 1:200 in BSA buffer) were incubated overnight at 4°C. Post-PBS washes, fluorophore-conjugated secondary antibodies were applied for 2 hours at RT. Nuclei were counterstained with DAPI for 10 min. Fluorescence signals were visualized and recorded with an Olympus fluorescence microscope, and ImageJ software was utilized for quantitative analysis of fluorescence intensity.

## Enzyme-linked immunosorbent assay (ELISA)

ELISA was conducted to measure the levels of inflammatory cytokines. The protein concentration of the samples was quantified using the BCA protein assay kit (P0010S, Beyotime, China), and the commercial ELISA kits (Elabscience, China) were used to measure the levels of IL-1β, IL-6, TNF-α, VEGF, TGF-β and CD31 in the extracted samples according to the manufacturer's instructions.

## Western blotting analysis

Proteins were separated using 10% SDS-polyacrylamide gel electrophoresis and subsequently transferred to cellulose nitrate membranes. To block the membranes, a 5% skim milk powder treatment was added for 2 hours, and the samples were incubated overnight at 4°C with primary antibodies. The next day, the membranes were treated with horseradish peroxidase-conjugated secondary antibodies. The detection of bound proteins was conducted using enhanced chemiluminescence (Biosharp, Hefei, China), and images were captured with a chemiluminescence imaging system (Tanon, Shanghai, China). GAPDH served as the internal reference protein, and quantitative analysis was conducted using ImageJ software.

## Quantitative real-time (qRT) PCR

Total RNA was isolated from wound tissues using the Qiagen RNeasy kit (Qiagen, Hilden, Germany), and complementary DNA (cDNA) was extracted with an RT-PCR kit (TaKaRa, Kusatsu, Japan). Quantitative real-time PCR was conducted using SYBR Premix Ex TaqTM II (Tli RNaseH Plus, TaKaRa). The relative expression levels of genes were calculated using the 2^−ΔΔCt^ method, normalized to those of GAPDH, and compared to the control group. The sequences of primers utilized are listed in **Table S1**.

## Statistical analysis

SPSS statistical software (version 21.0) was used for statistical analysis, and results were presented as the means ± SEMs. GraphPad Prism 8.3 was used to draw the graphs. Means were statistically compared using one-way ANOVA and the least significant difference (LSD) post hoc test, with *p< 0.05* considered statistically significant.

**Table S1.** Sequence of primers for quantitative PCR analysis

| Primer name | Sequence (5’-3’) |
| --- | --- |
| IL-1β-F | ACATCAACAAGAGCTTCAGG |
| IL-1β-R | AGGTGGAGAGCTTTCAGCTC |
| IL-6-F | CACAAGTCCGGAGAGGAGAC |
| IL-6-R | TCATCGTTGTTCATACAATC |
| TNF-α-F | CTCTTAATAGCAGGTCTATG |
| TNF-α-R | GTCAGCGGAAAGATTAGGAT |
| CD31-R | GTCCTGCTCCGTCTCGGGCA |
| CD31-F | TGGGCTTCGAGAGCATTTCG |
| TGF-β-F | GCGTGCTAATGGTGGACCGC |
| TGF-β-R | GCCAGGAATTGTTGCTATAT |
| VEGF-F | TCCACCATGCCAAGTGGTCC |
| VEGF-R | GCCTGCACAGCGCATCAGCG |
| NLRP3-F | TGTACGAGATGCAGGAGGAA |
| NLRP3-R | CCTGGTCCAAGGGTCGACCT |
| NF-κB-F | GGAAGCTGGATGATCCAGAG |
| NF-κB-R | CCATGCCAGATGTGGAGATG |
| IκBα-F | AGCTCGTCCGCGCCATGTTC |
| IκBα-R | CCATCTGCTCGTACTCCTCG |
| IL-18-F | GAAGAAAATGGAGACCTGGA |
| IL-18-R | TGCCAGTGAACCCCAGACCA |
